# Vulnerability, protection and fairness: ethical and regulatory limits in small-N gene therapy trials

**DOI:** 10.1186/s13063-026-09896-w

**Published:** 2026-07-11

**Authors:** Margaux Reckelbus, Eva Van Steijvoort, Isabelle Huys, Pascal Borry

**Affiliations:** 1 Department of Public Health and Primary Care, Centre for Biomedical Ethics and Law, KU Leuven, Kapucijnenvoer 7, Leuven, 3000 Belgium; 2 Department of Pharmaceutical and Pharmacological Sciences, KU Leuven, ON2 Herestraat 49, Leuven, 3000 Belgium; 3 Department of Human Genetics, ON6 Herestraat 49, Leuven, 3000 Belgium

**Keywords:** Gene therapy, Rare diseases, Clinical trial, Fair inclusion, Vulnerability, Procedural fairness

## Abstract

**Background:**

International ethics frameworks emphasize both the protection of vulnerable participants and the importance of equitable inclusion in research. In ultra-rare gene therapy trials, extensive safety monitoring, long-term follow-up requirements, and specialized treatment infrastructure are necessary to ensure participant protection and scientific validity. However, these requirements operate within healthcare systems characterized by geographic, financial, and social inequalities, raising concerns about whether all eligible patients have a fair opportunity to participate. Recent regulatory reforms in the European Union (EU), United Kingdom (UK), and United States (US) increasingly promote methodological flexibility for small patient populations through adaptive trial designs, risk proportionate oversight, and alternative evidentiary approaches.

**Main body:**

This commentary examines the extent to which existing clinical trial guidance for gene therapy promotes fair inclusion while safeguarding participants. We analyze binding and non-binding guidance from the European Medicines Agency (EMA), United States Food and Drug Administration (FDA), Medicines and Healthcare products Regulatory Agency (MHRA), International Council for Harmonisation (ICH), Council for International Organizations of Medical Sciences (CIOMS), and United Nations Educational, Scientific and Cultural Organization (UNESCO), alongside illustrative examples from Zolgensma®, Roctavian®, and Hemgenix®. Current frameworks provide substantial methodological flexibility with natural history data, external controls, decentralized approaches, and proportionate evidence requirements. Emerging initiatives such as FDA Diversity Action Plans, the UK Inclusion and Diversity Plan, and recent EU regulatory reforms further reflect growing attention to procedural fairness. Nevertheless, operational barriers remain. Long-term follow-up obligations, geographic concentration of expertise, financial burdens, and reliance on patient advocacy networks continue to shape who can realistically participate in research. While regulators increasingly recognize these challenges, guidance rarely requires concrete measures to mitigate them.

**Conclusion:**

Current gene therapy trial frameworks largely succeed in supporting methodological flexibility for ultra-rare conditions but insufficiently operationalize fair inclusion. Ethical concerns arise not because participant protections are excessive, but because the burdens associated with necessary scientific and regulatory requirements may disproportionately affect patients facing structural disadvantages. Ensuring justice in ultra-rare gene therapy research requires extending proportionate, risk-based approaches beyond evidence generation to include the identification and mitigation of foreseeable barriers to participation.

## Background

International ethics frameworks consistently emphasize that certain individuals and groups may require additional consideration in research contexts due to their specific circumstances. For example, the Universal Declaration on Bioethics and Human Rights states that human vulnerability should be taken into account and that individuals and groups in situations of vulnerability should receive appropriate protection and respect for their integrity [1]. Similarly, the Council for International Organizations of Medical Sciences (CIOMS) highlights that vulnerability should not be used as a basis for exclusion from research that may offer potential benefit, but rather as a basis for ensuring appropriate safeguards [2]. In this paper, vulnerability is understood in line with established bioethical frameworks as a context-dependent condition in which individuals or groups may face an increased likelihood of harm or reduced capacity to protect their interests due to medical, social, economic, or structural factors, particularly within research settings. Accordingly, vulnerability is not treated as a categorical basis for exclusion from research participation, but as a contextual ethical consideration relevant to the design and conduct of clinical trials [2, 3].

Recent scholarship has highlighted a “protection-inclusion dilemma” in research ethics, where Institutional Review Boards (IRBs) must navigate competing demands to protect participants from harm while ensuring diverse populations are included. Historically, a “history of protectionism” led to the systematic exclusion of groups perceived as vulnerable, such as women, children, and racialized populations. More recently, scholars have argued that this over-protection through exclusion constitutes both an epistemic shortcoming and an ethical failure, as it denies individuals the opportunity to contribute to science and unfairly excludes them from participating in research that may contribute to the development of novel treatments. Effective ethical oversight, therefore, requires IRBs to move beyond a default position of exclusion and instead strike a better balance by calibrating protections with actual risks. This tension is particularly salient in contexts where failing to include diverse participants results in significant gaps in health data and the generalizability of research findings [4]. Although concerns about over-protection and exclusion have been raised in the broader research ethics literature, the barriers to participation observed in ultra-rare gene therapy research often stem from the practical implications of scientific, regulatory, and logistical requirements [5, 6].

In ultra-rare gene therapy research, a range of regulatory and methodological requirements are designed to ensure participant safety and data validity. These include stringent consent procedures, risk minimization strategies, and long-term follow-up (LTFU) obligations to monitor delayed adverse effects. The US Food and Drug Administration (FDA), for example, emphasizes the importance of long-term observational studies for collecting data on delayed safety outcomes following gene therapy administration, which may extend over many years post-treatment. These requirements are primarily driven by scientific and regulatory objectives related to safety and efficacy assessment, rather than by vulnerability status per se [7, 8].

At the same time, logistical and structural challenges inherent to advanced therapies can significantly affect trial participation. These include centralized manufacturing processes, short product shelf-life, complex administration pathways, and the need for specialized follow-up infrastructure, all of which may limit participation for patients located far from expert centers or outside established care and advocacy networks [9–11]. Consequently, even when eligibility criteria are met, practical barriers may shape who is realistically able to participate in research. An ethical tension therefore arises not from vulnerability-based protections themselves, but from the interaction between necessary scientific and regulatory requirements and existing structural inequalities in access to care and research infrastructure in a system that prioritizes process equality over outcome equity [12].

Regulators have increasingly introduced flexibility to accommodate very small patients populations while maintaining scientific rigor. The European Medicines Agency (EMA) notes that, in the context of very rare diseases, “the combined evaluation of single case studies may be the only way to provide evidence” [13]. Similarly, FDA guidance on rare diseases highlights innovative trial designs, external controls, and natural history data to meet statutory standards with proportionate evidence when enrollment is constrained [14]. The International Council for Harmonisation (ICH) E6(R3) furthermore also embeds “a risk-based and proportionate approach” alongside Quality by Design as Good Clinical Practice (GCP) principles, thereby explicitly encouraging fit-for-purpose trial conduct that minimizes unnecessary burdens on participants and sites [8].

In the European Union (EU), recent and upcoming regulatory instruments are reshaping the design and evaluation of gene therapy trials. The EMA’s 2025 “Guideline on quality, non-clinical and clinical requirements for investigational advanced therapy medicinal products” (effective 1 July 2025) clarifies updated expectations for early-phase studies, risk assessment, and quality data for Advanced Therapy Medicinal Products (ATMP) [15]. In parallel, the Clinical Trials Regulation (EU No 536/2014) and its centralized Clinical Trials Information System (CTIS), mandatory for all new and ongoing trials as of 31 January 2025, aim to harmonize submissions, improve transparency, and ensure consistent regulatory oversight across Member States [16].

Furthermore, revisions to the European Pharmacopoeia, notably the new general monograph “Gene therapy medicinal products for human use (3186)” and supporting chapter 5.34, effective April 2025, establish standardized control and quality requirements for viral vector and transgene-based products [17]. Collectively, these updates signal a transition toward a more unified EU framework for ATMP regulation, one that enhances predictability and alignment, but also simultaneously increases procedural and compliance demands for sponsors and investigators.

In the United Kingdom (UK), ethical and regulatory oversight of gene therapy trials is coordinated through the Gene Therapy Advisory Committee (GTAC), which serves as the national Research Ethics Committee for gene therapy clinical research. Applications for ethical approval must be submitted via the Integrated Research Application System (IRAS) and are reviewed under the Medicines for Human Use (Clinical Trials) Regulations 2004 [18, 19]. The Medicines and Healthcare products Regulatory Agency (MHRA) is responsible for ensuring compliance with GCP standards, including risk-adapted approaches and the conduct of inspections [20]. Recent UK guidance emphasizes the importance of governance and operational readiness, highlighting the role of Genetic Modification Safety Committees, and provides direction on the preparation and delivery of gene therapies at point-of-care sites [21]. Importantly, the Health Research Authority (HRA)-MHRA Inclusion and Diversity Plan, currently in its pilot phase, aims to ensure that underserved populations are not excluded from research participation. Sponsors are encouraged to proactively develop strategies for inclusive recruitment and retention, particularly in rare disease trials [22].

However, flexible regulatory mechanisms alone do not guarantee equitable access. The European Commission’s ATMP-specific GCP guidance underlines the logistical complexity inherent to these products but stops short of mandating operational supports (e.g., travel funding, decentralized follow-up) that would convert flexibility into equitable participation [23]. Similarly, the FDA’s emerging policy on Diversity Action Plans signals a shift toward embedding inclusion planning early in development, outlining “the form, content, and manner of diversity action plans…” [24]. Yet, how these expectations apply to ultra-rare gene therapy remains insufficiently specified in binding regulatory terms.

This paper argues that while current guidance provides adequate methodological flexibility for small-N gene therapy trials, it insufficiently operationalizes the principle of fair inclusion. Participant protection remains non-negotiable; the central challenge lies in achieving inclusion while ensuring that protective requirements do not disproportionately burden patients who already face geographic, financial, or social barriers to participation.

We therefore ask: to what extent do existing clinical trial guidelines for gene therapy promote fair inclusion of rare disease populations while protecting vulnerable patients? We analyze binding and non-binding guidance across the EU, UK and United States (US) encompassing instruments from the International Council for Harmonisation of Technical Requirements for Pharmaceuticals (ICH), CIOMS and United Nations Educational, Scientific and Cultural Organization (UNESCO), with particular attention to recruitment practices in small-N gene therapy, while recognizing that early phase uncertainty, cross-border access, and LTFU obligations shape fairness on the ground. Through cases studies of licensed products (e.g., Zolgensma®, Roctavian®, Hemgenix®) we aim to illustrate how design flexibility can advance equity, while identifying the persistent regulatory and ethical gaps that constrain it.

## Main text

### Defining fairness in ultra-rare contexts

In this paper, fair inclusion does not imply a right to participate in research nor an obligation to eliminate all barriers to participation. Rather, it refers to the ethical requirement that recruitment, enrollment, and long-term follow-up processes should not systematically disadvantage otherwise eligible participants on the basis of remediable social, economic, geographic, or structural barriers. This conception draws on CIOMS guidance concerning unjust exclusion and equitable participant selection, which emphasizes that inclusion and exclusion criteria should not be based on discriminatory or ethically irrelevant factors without adequate justification, as well as emerging scholarship on the protection-inclusion dilemma in research ethics [2, 3, 25]. Accordingly, the focus is not on guaranteeing equal participation outcomes, but on ensuring that avoidable barriers do not systematically determine who can realistically access research opportunities.

For diseases with extremely small patient populations, randomized controlled trials (RCTs) are often infeasible. Regulators such as the EMA, FDA, and MHRA have acknowledged this constraint by permitting alternative forms of evidence, including case series, external comparators, and the use of natural history data. Under the EMA’s 2025 ATMP guideline, such flexible evidentiary approaches are formally recognized for early-phase clinical development, provided that associated risks and uncertainties are transparently addressed [15]. Similarly, the UK’s Medicines for Human Use (Clinical Trials) (Amendment) Regulations 2024, set to take full effect in April 2026, promote a risk-proportionate and flexible regulatory environment designed to support innovation in rare disease trials [26]. While these forms of flexibility are vital for advancing therapies for ultra-rare conditions toward regulatory approval, they do not in themselves guarantee equitable access to trial participation. Achieving fairness in practice requires what may be termed access parity: the genuine possibility for all eligible patients to participate, regardless of geography, socio-economic status, or disease subtype [9, 27, 28].

Although access to research participation and access to treatment are conceptually distinct, the distinction becomes less clear in the context of ultra-rare gene therapy. For many patients, clinical trials may represent the primary or only route through which investigational therapies can be accessed. This paper focuses on fairness in research participation rather than entitlement to treatment. Even so, because opportunities for participation may simultaneously function as opportunities for access to investigational therapies, the ethical implications of inclusion decisions warrant particular attention [29].

This growing regulatory openness reflects a broader shift in how ultra-rare diseases are approached. The EMA has acknowledged that: “In very rare diseases, the combined evaluation of single case studies may be the only way to provide evidence” [13]. Similarly, the FDA’s Rare Diseases guidance affirms that the Agency will exercise “flexibility” in meeting evidentiary standards, encouraging the use of innovative trial designs and external controls where appropriate [30]. The ICH, through its GCP revision E6(R3), likewise embeds the principles of proportionality and pragmatism within clinical research oversight [8]. The UK’s MHRA aligns with these principles, emphasizing adaptive trial designs, decentralized methodologies, and streamlined approvals for rare disease trials [20]. Complementing these efforts, the National Institute for Health and Care Excellence (NICE) Highly Specialised Technologies (HST) programme further supports this by applying higher cost-effectiveness thresholds and flexible evidence requirements for ultra-rare conditions [31].

Decentralized trial methods encourage remote participation, telemedicine, and home-based assessments [32–34]. These approaches are particularly valuable for patients with rare diseases who may face mobility or geographic constraints [35–37]. Methodological concessions such as natural history comparators not only facilitate the feasibility of ultra-rare studies but also mitigate the ethical concern of exposing medically fragile patients to placebo assignments that may be clinically non-beneficial or ethically problematic in contexts of progressive or severe disease [38]. At the same time, these approaches involve important trade-offs regarding evidentiary robustness and the generation of generalizable knowledge, which regulators must carefully balance [39]. Moreover, the forthcoming EU CTIS framework and the UK’s IRAS both aim to enhance cross-border trial visibility and coordination [19, 40].

Another methodological approach receiving increasing attention in rare disease research is the use of Single-Case Experimental Designs (SCEDs) and N-of-1 trials. These designs may be particularly valuable in ultra-rare conditions where patient populations are extremely limited and substantial clinical heterogeneity exists [41]. Unlike conventional group-based trials, SCEDs allow participants to serve as their own controls, thereby reducing the influence of inter-individual variability and enabling more individualized assessments of treatment response [42]. From an ethical perspective, these designs can support fair inclusion. By maximizing the information gained from each participant and reducing the need for large recruitment targets, they may be particularly well suited to ultra-rare populations in which conventional enrollment requirements are often unattainable [43]. They are also well aligned with the goals of precision medicine, as they focus on determining whether an intervention is effective for a particular patient rather than solely assessing average treatment effects across a population [44]. Furthermore, evidence generated through multiple single-case studies can be systematically combined to support broader conclusions regarding safety and effectiveness [41]. Although SCEDs are not suitable for every gene therapy context, particularly where irreversible interventions limit repeated treatment comparisons, they illustrate how methodological innovation can simultaneously advance scientific validity, participant protection, and equitable access to research opportunities.

Despite regulatory openness, operational barriers continue to stratify access. In practice, only those with sufficient resources, proximity to trial centers, or robust institutional support are positioned to take advantage of these research opportunities [45, 46]. Thus, while formal conditions for inclusion exist, operational realities often stratify access. Even well-intentioned reforms risk entrenching disparities if smaller sponsors lack the resources to meet documentation or quality requirements [47, 48].

An example is the approval of Zolgensma® for spinal muscular atrophy (SMA). The therapy was authorized based on a single-arm clinical trial contextualized by natural history comparators. According to the FDA Summary Basis for Regulatory Action, the Agency accepted that comparison to “available natural history data” provided sufficient primary evidence of effectiveness [49]. This approach avoided the ethical dilemma of randomizing critically ill infants to a placebo arm while still generating persuasive evidence of therapeutic benefit [50]. Yet, recruitment remained limited to specialized centers, showing that regulatory flexibility cannot alone overcome resource or geographic barriers [49].

Achieving fairness in practice requires additional scaffolding (logistical, financial, and organizational). Without mechanisms to subsidize travel and accommodation, support caregivers, or facilitate decentralized and cross-border participation, disparities in trial access persist. Trial designs that are inclusive in principle but operationally inaccessible risk perpetuating inequality by privileging those with the means to surmount these logistical barriers. Justice in research thus demands not only methodological flexibility but structural supports that make participation feasible for all eligible patients [51–53].

### Expanding inclusion

Too often, diversity and access considerations are treated as secondary concerns, addressed only after recruitment challenges emerge [51, 54]. This reactive approach risks perpetuating entrenched inequities: patients with financial resources, mobility, and well-connected physicians are overrepresented, while those facing geographic, caregiving, or language-related barriers remain underrepresented or excluded [55–58].

Recent policy developments indicate a shift toward structural planning. The FDA’s draft guidance on Diversity Action Plans seeks to move from ad hoc outreach to systematic, up-front commitments: “This draft guidance describes the form, content, and manner of diversity action plans” [24]. The guidance proposes that sponsors define enrollment targets and retention strategies prior to trial initiation. By encouraging structured planning for diversity, these plans are intended to be considered within regulatory review processes, positioning inclusion as a more measurable component of clinical research [59, 60]. This framework has not been finalized as binding guidance and its implementation remains uncertain, meaning that its practical regulatory effect is currently limited. However, it reflects an ongoing policy direction toward more structured attention to diversity and inclusion in clinical trial design.

In the UK, the HRA and MHRA have jointly developed an Inclusion and Diversity Plan framework, currently being piloted with researchers and sponsors [22]. This initiative encourages sponsors to proactively address barriers faced by underserved populations during trial design, including caregiving responsibilities, language differences, and geographic isolation. It is intended to become a standard part of trial applications, reinforcing the UK’s commitment to inclusive research [22, 61].

In Europe, the ATMP-GCP guideline explicitly acknowledges the distinctive logistical burdens associated with gene therapy trials, such as “manufacturing constraints and the short shelf-life of the product” and the need for “specific arrangements for long-term follow-up” [23].While this recognition signals regulatory awareness of patient and site burdens, the guidance stops short of prescribing concrete remedies. The forthcoming EMA guideline and CTIS integration build on this foundation, signaling that inclusion and proportionality are increasingly recognized as central to clinical development strategy, even though these expectations remain non-binding in operational terms [40, 62].

The ICH E6(R3) revision sets out a flexible and proportionate framework, specifying “a risk-based and proportionate approach to the conduct of a clinical trial” [8]. This principle logically extends to fairness: if inequitable inclusion threatens scientific validity or ethical legitimacy, then safeguards (such as funded travel, remote assessments, and translation services) ought to be mandated as part of quality-by-design. The MHRA has confirmed that ICH E6(R3) will be implemented alongside revised UK clinical trial regulations by mid-2026, reinforcing proportionality and procedural fairness as formal regulatory expectations [63].

Despite these positive signals, current guidance leaves considerable discretion to sponsors. In the absence of binding requirements, trial sites frequently default to convenience-based recruitment [64, 65]. In ultra-rare diseases, where each participant counts and available enrollment slots may be as few as ten or twenty, exclusions on financial or logistical grounds are not incidental, they shape access to potentially curative interventions and can undermine both justice and scientific validity [46, 66–68].

A global trend is emerging toward embedding proportionality and inclusivity into regulatory expectations [69, 70]. In the UK, the England Rare Diseases Action Plan 2025 emphasizes equitable access to specialist care and clinical trials, introducing measures aimed at reducing geographic and socio-economic barriers to participation [71].

Equity in research participation is fundamentally a matter of justice. Patients with ultra-rare conditions already face severely limited therapeutic options; excluding some on logistical or socio-economic grounds compounds their disadvantage [72]. Moreover, representativeness in small-N studies is a necessity: when trials recruit only the most affluent or mobile patients, the resulting evidence may fail to reflect the broader patient population [73–75].

The shift toward procedural fairness carries ethical, scientific, and political significance. Ethically, it ensures that research opportunities are distributed justly [62]. Scientifically, it strengthens the generalizability and validity of trial findings. Politically, it aligns with the growing international consensus, including the UK’s evolving regulatory framework, that diversity and proportionality must be embedded from the outset of clinical research [22, 76].

### The role and risk of advocacy networks in ultra-rare gene therapy trials

Patient advocacy networks play a uniquely important role in ultra-rare diseases. In conditions where total patient numbers may be measured in dozens or hundreds, traditional recruitment pathways often prove insufficient. Advocacy organizations provide visibility, community support, and access to patient lists that may otherwise be impossible for sponsors to compile [77, 78]. Yet these strengths give rise to risks when advocacy networks become the primary conduit for trial access. Over-reliance on advocacy-mediated recruitment can produce a skewed trial population disproportionately composed of well-connected, internet-literate, and socially mobile patients. Those already embedded in advocacy circles are more likely to learn of research opportunities, while patients outside these networks risk systematic exclusion [79, 80].

CIOMS Guideline 3 states: “The equitable distribution of benefits and burdens in the selection of individuals and groups of participants in research is a fundamental requirement of just research conduct” [2]. The mandate is clear: opportunities must not reinforce existing inequalities. However, while the principle is well established, operational guidance remains limited.

The UK’s Inclusion and Diversity Plan, jointly developed by the MHRA and HRA, explicitly calls for inclusive recruitment strategies that address barriers faced by underserved populations. Sponsors are instructed to consider socio-economic, geographic, and cultural factors when designing outreach and enrollment procedures [22]. Complementing this, the National Institute for Health and Care Research (NIHR) INCLUDE framework further supports this by offering practical tools to embed equity, diversity, and inclusion (EDI) throughout the research lifecycle [81].

Existing guidelines generally address equity in general terms but stop short of providing operational mechanisms [51]. Furthermore, research systems often lack the accountable frameworks required to overcome the ethical and technical challenges inherent in ensuring trial conduct is accessible and representative [76]. The result is a fairness vacuum: everyone agrees equity matters, yet no one prescribes procedures to guarantee it.

Advocacy networks are indispensable allies, but reliance without safeguards risks over-representation of the well-connected and exclusion of the less-connected patients. Bridging this gap requires moving from aspirational fairness to procedural fairness [82].

Within the EU’s updated framework, procedural fairness could be embedded as a “critical-to-quality” element under CTIS oversight, requiring sponsors to disclose recruitment strategies and accessibility measures in trial applications [83]. In the UK, the 2025 Clinical Trials Regulations and England Rare Diseases Action Plan similarly emphasize the need for transparent, inclusive, and community-informed trial design [84]. Collectively, these reforms aim to position the UK a global leader in equitable clinical research, particularly for rare and ultra-rare conditions.

### Ethical limits in small-N trials

The ethical complexity of ultra-rare gene therapy trials derives not only from the small patient populations but also from the irreversible nature of the interventions. Gene therapies are often administered as a single, one-time intervention designed to confer durable or potentially lifelong effects. This “one-shot” characteristic distinguishes them from conventional therapies, where patients may enroll in successive trials or discontinue treatment if adverse effects emerge [85].

The EMA’s 2025 guideline formalizes expectations for LTFU data collection and safety monitoring for investigational ATMPs, emphasizing proportionate approaches based on identified risks [86, 87]. Similarly, the UK’s MHRA requires risk-based LTFU protocols for gene therapy trials highlighting the collection of durability, safety, and real-world evidence [88]. Ethical oversight is ensured by the HRA GTAC for irreversible interventions [89]. In parallel, the FDA recommends designing long-term observational studies, noting: “We provide recommendations regarding the design of long-term follow-up studies (LTFU observations) for the collection of data on delayed adverse events following administration of a gene therapy product” [7], and the European Commission likewise notes that “the long-term effects of the product may require specific arrangements for long-term follow-up of the subjects” [23].

For patients and families, enrollment carries a dual weight: once administered, the patient may be precluded from accessing future, improved therapies and is simultaneously committed to LTFU obligations. When these obligations extend over years or decades, the burdens can become asymmetrical. Ethical inclusion therefore demands practical supports and methodological approaches that minimize the opportunity costs of participation [90–92].

The potential exclusionary effects of protective measures rarely arise from the safeguards themselves, but rather from the burdens associated with complying with them. Long-term follow-up requirements are ethically justified because of the uncertainty surrounding delayed gene therapy risks. However, when follow-up requires repeated travel to specialist centers, time away from work, or sustained caregiver involvement, these requirements may disproportionately affect patients with fewer resources [93]. Fair inclusion therefore requires attention not only to the necessity of safeguards but also to how their burdens are distributed.

Illustrative examples include Roctavian®, for hemophilia A, which relied on non-randomized evidence using within-subject comparisons to measure outcomes before and after treatment, with LTFU to assess durability and safety [94, 95]. Hemgenix®, for hemophilia B, followed a similar pathway [96]. These designs illustrate how methodological choices in ultra-rare gene therapy trials can support both scientific validity and fair inclusion of patients in evidence generation. By avoiding traditional placebo-controlled designs and instead relying on within-subject or natural history comparators, these approaches reduce the likelihood that patients are excluded from participation or assigned to non-therapeutic arms where no meaningful clinical benefit would be expected. Such methodological adaptations therefore help align evidentiary requirements with ethical considerations of inclusion in ultra-rare, high-stakes contexts, where each participant represents a significant proportion of the patient population. In this sense, trial design itself can function as a mechanism that supports more equitable opportunities for participation in research [38, 82, 97, 98].

Nevertheless, LTFU obligations often impose disproportionate burdens, travel, income loss, caregiver strain [46, 99–101]. As affluent families are more able to sustain long-term commitments, participation may become a function of wealth, undermining both ethical legitimacy and scientific validity[72, 102].

Achieving meaningful fairness therefore requires interventions beyond formal eligibility. Sponsor-funded LTFU supports can prevent participation from being contingent on patient wealth. Moreover, methodological designs that minimize opportunity costs ensure that participation does not necessitate ethically untenable compromises.

These measures align with the proportionate, risk-based approach in ICH E6(R3): “This guideline encourages a risk-based and proportionate approach to the conduct of a clinical trial” [8]. Just as quality-by-design safeguards data integrity and patient safety, it should also incorporate mechanisms to mitigate fairness risks [103, 104]. The UK’s Rare Diseases Framework and England Rare Diseases Action Plan 2025 further emphasize the need for equitable access to specialist treatments, including support for long-term care and follow-up [71, 105].

The obligations proposed here should not be understood as unlimited. Fair inclusion does not require sponsors to eliminate every conceivable barrier to participation. Rather, consistent with the proportionality principles embedded in ICH E6(R3) [8], sponsors should be expected to identify and mitigate foreseeable and remediable barriers that could systematically exclude otherwise eligible participants. The adequacy of these measures will necessarily depend on factors such as trial burden, available resources, sponsor capacity, and the specific needs of the study population. Fair inclusion should therefore be understood as a proportionate objective that requires balancing accessibility, scientific validity, participant understanding, and operational feasibility.

## Conclusion

Ultra-rare gene therapy trials test the limits of traditional research ethics. In these contexts, every participant represents a meaningful fraction of the total patient population, and every procedural choice (from consent to follow-up) has amplified ethical and scientific consequences. Current international and national frameworks (UNESCO, CIOMS, ICH, EMA, FDA, MHRA) rightly emphasize protection, proportionality, and methodological flexibility. Yet flexibility without enforceable operational supports risks entrenching inequity. The challenge identified in this paper is not that participant protections are excessive, but that necessary protective requirements often operate within healthcare systems marked by geographic, financial, and social inequalities. When inclusion is left to the discretion of sponsors or the resourcefulness of patients, participation risks becoming dependent on privilege rather than on equitable opportunity to participate.

As this analysis shows, regulators have made substantial progress: the EMA’s 2025 ATMP guideline, the EU’s CTIS integration, the UK’s Inclusion and Diversity Plan, and the FDA’s Diversity Action Plans all recognize that fairness cannot be an afterthought. However, the operational translation of this principle remains incomplete. Guidance documents frequently commend inclusion but rarely mandate concrete supports (such as travel assistance, decentralized assessments, or caregiver compensation) that would make participation feasible for all eligible patients. This omission shifts the burden of fairness from institutions to individuals, undermining the very principles of justice and solidarity these frameworks seek to uphold.

Ensuring fairness in ultra-rare gene therapy trials thus requires moving beyond methodological flexibility toward structural equity. Proportionate approaches must extend not only to evidence generation but also to access and follow-up. Ethical inclusion demands that logistical and economic barriers be treated as risks to trial quality, risks that must be mitigated by design rather than managed informally. Integrating fairness as a “critical-to-quality” element within regulatory oversight (e.g., under CTIS or MHRA review) would ensure that inclusion plans, patient supports, and LTFU accommodations are scrutinized alongside scientific validity.

Ultimately, procedural fairness is an ethical necessity. It aligns the principle of protection with meaningful participation, ensuring that safeguards and regulatory requirements do not, in practice, interact with structural and logistical constraints in ways that disproportionately limit access to research participation. In the context of ultra-rare gene therapy, justice requires more than access to treatment; it requires access to the opportunity to participate in research itself.

## Data Availability

No datasets were generated or analysed during the current study.

## Abbreviations

ATMP: Advanced therapy medicinal products
CIOMS: Council for International Organizations of Medical Sciences
CTIS: Clinical Trials Information System
EMA: European Medicines Agency
EU: European Union
FDA: The U.S. Food and Drug Administration
GCP: Good Clinical Practice
GTAC: Gene Therapy Advisory Committee
HRA: Health Research Authority
HST: Highly specialised technologies
ICH: International Council for Harmonisation of Technical Requirements for Pharmaceuticals
IRAS: Integrated Research Application System
LTFU: Long-term follow-up
MHRA: Medicines and Healthcare products Regulatory Agency
NICE: National Institute for Health and Care Excellence
NIHR: National Institute for Health and Care Research
RCT: Randomized controlled trials
SMA: Spinal muscular atrophy
UK: United Kingdom
UNESCO: United Nations Educational, Scientific and Cultural Organization
US: United States
WHO: World Health Organization

## References

[1] Universal declaration on bioethics and human rights - legal affairs. https://www.unesco.org/en/legal-affairs/universal-declaration-bioethics-and-human-rights. Accessed 2 Oct 2025.

[2] Council for International Organizations of Medical Sciences (CIOMS). International ethical guidelines for health-related research involving humans. Council for International Organizations of Medical Sciences (CIOMS). 2016. 10.56759/rgxl7405. 40523065

[3] Bracken-Roche D, Bell E, Macdonald ME, Racine E. The concept of ‘vulnerability’ in research ethics: an in-depth analysis of policies and guidelines. Health Res Policy Syst. 2017;15:8. 10.1186/s12961-016-0164-6. 28173859 10.1186/s12961-016-0164-6 PMC5297186

[4] Friesen P, Gelinas L, Kirby A, Strauss DH, Bierer BE. IRBs and the protection-inclusion dilemma: finding a balance. Am J Bioeth. 2023;23:75–88. 10.1080/15265161.2022.2063434. 35482887 10.1080/15265161.2022.2063434 PMC9926358

[5] Levine C, Faden R, Grady C, Hammerschmidt D, Eckenwiler L, Sugarman J. The limitations of “vulnerability” as a protection for human research participants. Am J Bioeth. 2004;4:44–9. 10.1080/15265160490497083. 16192138 10.1080/15265160490497083

[6] Rath A, Salamon V, Peixoto S, Hivert V, Laville M, Segrestin B, et al. A systematic literature review of evidence-based clinical practice for rare diseases: what are the perceived and real barriers for improving the evidence and how can they be overcome? Trials. 2017;18:556. 10.1186/s13063-017-2287-7. 29166947 10.1186/s13063-017-2287-7 PMC5700662

[7] U.S. Department of Health and Human Services, Food and Drug Administration, Center for Biologics Evaluation and Research. Long Term Follow-Up After Administration of Human Gene Therapy Products: Guidance for Industry. Silver Spring (MD): Food and Drug Administration; 2020. Available from: https://www.fda.gov/media/113768/download. Accessed 2 Oct 2025.

[8] Research C for DE and. E6(R3) Good Clinical Practice (GCP). 2025. https://www.fda.gov/regulatory-information/search-fda-guidance-documents/e6r3-good-clinical-practice-gcp. Accessed 3 Oct 2025.

[9] Sava J. Addressing the barriers to equitable access in cell and gene therapies | Targeted oncology - immunotherapy, biomarkers, and cancer pathways. 2025. https://www.targetedonc.com/view/addressing-the-barriers-to-equitable-access-in-cell-and-gene-therapies. Accessed 2 Oct 2025.

[10] Exploring barriers to accessing gene and cell therapy treatments. ASGCT. https://www.asgct.org/news-publications/asgct-news/exploring-barriers-to-accessing-gene-and-cell-therapy-treatments. Accessed 2 Oct 2025.

[11] Addressing gene therapy’s ethical and policy challenges. The hastings center for bioethics. 2024. https://www.thehastingscenter.org/news/addressing-gene-therapys-ethical-and-policy-challenges/. Accessed 2 Oct 2025.

[12] Warner E, Marron JM, Peppercorn JM, Abel GA, Hantel A. Shifting from equality toward equity: addressing disparities in research participation for clinical cancer research. J Clin Ethics. 2024;35:8–22. 10.1086/728144. 38373334 10.1086/728144 PMC10983799

[13] Clinical trials in small populations - Scientific guideline | European Medicines Agency (EMA). 2006. https://www.ema.europa.eu/en/clinical-trials-small-populations-scientific-guideline. Accessed 3 Oct 2025.

[14] Research C for DE and. Rare diseases: considerations for the development of drugs and biological products. 2024. https://www.fda.gov/regulatory-information/search-fda-guidance-documents/rare-diseases-considerations-development-drugs-and-biological-products. Accessed 3 Oct 2025.

[15] Guideline on quality, non-clinical and clinical requirements for investigational advanced therapy medicinal products in clinical trials - Scientific guideline | European Medicines Agency (EMA). 2019. https://www.ema.europa.eu/en/guideline-quality-non-clinical-clinical-requirements-investigational-advanced-therapy-medicinal-products-clinical-trials-scientific-guideline. Accessed 3 Oct 2025.

[16] Clinical Trials Regulation | European Medicines Agency (EMA). 2015. https://www.ema.europa.eu/en/human-regulatory-overview/research-development/clinical-trials-human-medicines/clinical-trials-regulation. Accessed 3 Oct 2025.

[17] New European Pharmacopoeia Commission approach to gene therapy - European Directorate for the Quality of Medicines & HealthCare - EDQM. European Directorate for the Quality of Medicines & HealthCare. https://www.edqm.eu/en/-/new-european-pharmacopoeia-commission-approach-to-gene-therapy-1. Accessed 3 Oct 2025.

[18] Gene Therapy Advisory Committee. Health Research Authority. https://www.hra.nhs.uk/about-us/committees-and-services/res-and-recs/gene-therapy-advisory-committee/. Accessed 3 Oct 2025.

[19] Integrated Research Application System. Health Research Authority. https://www.hra.nhs.uk/about-us/committees-and-services/integrated-research-application-system/. Accessed 3 Oct 2025.

[20] Risk-adapted approach to clinical trials and risk assessments. GOV.UK. https://www.gov.uk/government/publications/risk-adapted-approach-to-clinical-trials-and-risk-assessments. Accessed 3 Oct 2025.

[21] MHRA Inspectorate – News and updates from the MHRA Inspectorate. https://mhrainspectorate.blog.gov.uk/. Accessed 3 Oct 2025.

[22] Developing our inclusion and diversity plan and supporting guidance. Health Research Authority. https://www.hra.nhs.uk/planning-and-improving-research/best-practice/increasing-diversity-people-taking-part-research/developing-our-inclusion-and-diversity-plan-and-supporting-guidance/. Accessed 3 Oct 2025.

[23] Guidelines on good clinical practice specific to advanced therapy medicinal products - ECA Academy. https://www.gmp-compliance.org/guidelines/gmp-guideline/guidelines-on-good-clinical-practice-specific-to-advanced-therapy-medicinal-products. Accessed 3 Oct 2025.

[24] Commissioner O of the. Diversity action plans to improve enrollment of participants from underrepresented populations in clinical studies. 2025. https://www.fda.gov/regulatory-information/search-fda-guidance-documents/diversity-action-plans-improve-enrollment-participants-underrepresented-populations-clinical-studies. Accessed 3 Oct 2025.

[25] Emanuel EJ, Wendler D, Grady C. What makes clinical research ethical? JAMA. 2000;283:2701–11. 10.1001/jama.283.20.2701. 10819955 10.1001/jama.283.20.2701

[26] Clinical trials regulations signed into law. GOV.UK. https://www.gov.uk/government/news/clinical-trials-regulations-signed-into-law. Accessed 3 Oct 2025.

[27] Bloomfield LW Adam. Premier research | Flexibility drives success in rare disease trials. Premier Research. 2025. https://premier-research.com/perspectives/flexibility-drives-success-in-rare-disease-trials/. Accessed 9 Oct 2025.

[28] Accelerating global access to gene therapies: case studies from low- and middle-income countries. World Economic Forum. https://www.weforum.org/publications/accelerating-global-access-to-gene-therapies-case-studies-from-low-and-middle-income-countries/. Accessed 9 Oct 2025.

[29] Bateman-House A, Shah LD, Escandon R, McFadyen A, Hunt C. Somatic gene therapy research in pediatric populations: ethical issues and guidance for operationalizing early phase trials. Pharm Med. 2023;37:17–24. 10.1007/s40290-022-00451-x. 10.1007/s40290-022-00451-x 36527677

[30] Research C for DE and. Guidance documents for rare disease drug development. FDA. 2025.

[31] Changes approved to the routing criteria for highly specialised technologies. NICE website: The National Institute for Health and Care Excellence. 2025. https://www.nice.org.uk/news/articles/changes-approved-to-the-routing-criteria-for-highly-specialised-technologies. Accessed 3 Oct 2025.

[32] FDA outlines new review pathway for drugs treating ultra-rare diseases | BioPharma Dive. https://www.biopharmadive.com/news/fda-rare-disease-evidence-principles-drug-reviews/759237/. Accessed 3 Oct 2025.

[33] McKenzie 6 min read | Heather. FDA’s marks advocates for flexibility in rare disease gene therapy trials. BioSpace. 2024. https://www.biospace.com/fda-s-marks-advocates-for-flexibility-in-rare-disease-gene-therapy-trials. Accessed 3 Oct 2025.

[34] New FDA program adds flexibility for ultra-rare disease submissions. https://www.clinicalleader.com/doc/new-fda-program-adds-flexibility-for-ultra-rare-disease-submissions-0001. Accessed 3 Oct 2025.

[35] Deliver decentralised clinical trials in the UK | NIHR. https://www.nihr.ac.uk/support-and-services/industry/spotlights/decentralised-clinical-trials. Accessed 3 Oct 2025.

[36] MHRA guidance on decentralized clinical trials in the UK – clinical research made simple. 2025. https://www.clinicalstudies.in/mhra-guidance-on-decentralized-clinical-trials-in-the-uk/. Accessed 3 Oct 2025.

[37] Decentralised trial methods position statement. Health Research Authority. https://www.hra.nhs.uk/planning-and-improving-research/policies-standards-legislation/clinical-trials-investigational-medicinal-products-ctimps/decentralised-trial-methods-position-statement/. Accessed 3 Oct 2025.

[38] Bratton E, Holt C, Kromrey S, Keefer J. Natural history studies for rare diseases: development strategies for external comparator arms leveraging real world insights. White paper. Durham (NC): IQVIA; 2020. Available from: https://www.iqvia.com/-/media/iqvia/pdfs/library/white-papers/natural-history-studies-for-rare-diseases.pdf. Accessed 3 Oct 2025.

[39] Khachatryan A, Read SH, Madison T. External control arms for rare diseases: building a body of supporting evidence. J Pharmacokinet Pharmacodyn. 2023;50:501–6. 10.1007/s10928-023-09858-8. 37095406 10.1007/s10928-023-09858-8 PMC10673956

[40] Clinical Trials Information System | European Medicines Agency (EMA). 2022. https://www.ema.europa.eu/en/human-regulatory-overview/research-development/clinical-trials-human-medicines/clinical-trials-information-system. Accessed 3 Oct 2025.

[41] Hawksworth O, Chatters R, Julious S, Cook A, Biggs K, Solaiman K, et al. A methodological review of randomised n-of-1 trials. Trials. 2024;25:263. 10.1186/s13063-024-08100-1. 38622638 10.1186/s13063-024-08100-1 PMC11020886

[42] Taoui S, Lepage B, Marx M. Single-case experimental designs and N-of-1 trials—an alternative to randomized clinical trials for clinical research in otorhinolaryngology. JAMA Otolaryngol Head Neck Surg. 2023;149:767–8. 10.1001/jamaoto.2023.1578. 37535385 10.1001/jamaoto.2023.1578

[43] Defelippe VM, JMW van Thiel G, Otte WM, Schutgens REG, Stunnenberg B, Cross HJ, et al. Toward responsible clinical n-of-1 strategies for rare diseases. Drug Discov Today. 2023;28:103688. 10.1016/j.drudis.2023.103688. 10.1016/j.drudis.2023.103688 37356616

[44] NatesanBatley P, McClure EB, Brewer B, Contractor AA, Batley NJ, Hedges LV, et al. Evidence and reporting standards in N-of-1 medical studies: a systematic review. Transl Psychiatry. 2023;13:263. 10.1038/s41398-023-02562-8. 37463877 10.1038/s41398-023-02562-8 PMC10354076

[45] Strategic solutions for overcoming patient travel barriers in clinical trials. 2025. https://explore.scoutclinical.com/blog/clinical-trial-patient-travel-support. Accessed 3 Oct 2025.

[46] Cramer G. Reducing barriers to participation in clinical trials for rare diseases. ACRP. 2021. https://acrpnet.org/2021/08/16/reducing-barriers-to-participation-in-clinical-trials-for-rare-diseases. Accessed 3 Oct 2025.

[47] Coalition for Reducing Bureaucracy in Clinical Trials. Recommendations by the Coalition for Reducing Bureaucracy in Clinical Trials. Brussels: Coalition for Reducing Bureaucracy in Clinical Trials; 2021. Available from: https://bureaucracyincts.eu/. Accessed 3 Oct 2025.

[48] How Brexit could make or break UKs clinical research industry by 2025. CCRPS Clinical Research Taininrg. https://ccrps.org/clinical-research-blog/how-brexit-could-make-or-break-uks-clinical-research-industry-by-2025. Accessed 3 Oct 2025.

[49] Byrnes A. Summary basis for regulatory action: Zolgensma (onasemnogene abeparvovec-xioi). Silver Spring (MD): U.S. Food and Drug Administration, Center for Biologics Evaluation and Research; 2019. Available from: https://www.fda.gov/media/127961/download. Accessed 3 Oct 2025.

[50] Farrar MA, Calotes-Castillo L, De Silva R, Barclay P, Attwood L, Cini J, et al. Gene therapy-based strategies for spinal muscular atrophy—an Asia-Pacific perspective. Mol Cell Pediatr. 2023;10:17. 10.1186/s40348-023-00171-5. 37964159 10.1186/s40348-023-00171-5 PMC10645685

[51] Ozota GO, Chang R, Seoane-Vazquez E, Brown L. HPR11 diversity, equity, and inclusion in gene and cell therapies clinical trials. Value Health. 2025;28:S195–6. 10.1016/j.jval.2025.04.735.

[52] Lee TL, Sawai T. Navigating equity in global access to genome therapy expanding access to potentially transformative therapies and benefiting those in need requires global policy changes. Front Genet. 2024;15. 10.3389/fgene.2024.1381172. 10.3389/fgene.2024.1381172 PMC11024294 38638119

[53] Sava J. Addressing the barriers to equitable access in cell and gene therapies | Targeted oncology - immunotherapy, biomarkers, and cancer pathways. 2025. https://www.targetedonc.com/view/addressing-the-barriers-to-equitable-access-in-cell-and-gene-therapies. Accessed 3 Oct 2025.

[54] Diversity. CTTI. https://ctti-clinicaltrials.org/about/ctti-projects/diversity/. Accessed 3 Oct 2025.

[55] Alarcón Garavito GA, Gilchrist K, Ciurtin C, Khanna S, Chambers P, McNally N, et al. Enablers and barriers of clinical trial participation in adult patients from minority ethnic groups: a systematic review. Trials. 2025;26:65. 10.1186/s13063-025-08769-y. 39984940 10.1186/s13063-025-08769-y PMC11846389

[56] Medical G. Diversity in clinical trials: through the lens of genetics. Genome Medical. 2023. https://www.genomemedical.com/genetic-research-trials/diversity-in-clinical-trials/. Accessed 3 Oct 2025.

[57] Cramer G. Barriers to clinical trial enrollment: focus on underrepresented populations. ACRP. 2024. https://acrpnet.org/2024/04/12/barriers-to-clinical-trial-enrollment-focus-on-underrepresented-populations. Accessed 3 Oct 2025.

[58] Mishra S. language barriers in clinical trials participation: can innovation bridge gaps & improve diversity? TrialX. 2022. https://trialx.com/language-barriers-in-clinical-trials-participation-can-innovation-bridge-the-gaps-improve-diversity/. Accessed 3 Oct 2025.

[59] FDA reveals diversity action plan guide for clinical trials. pharmaphorum. 2024. https://pharmaphorum.com/news/fda-reveals-diversity-action-plan-guide-clinical-trials. Accessed 3 Oct 2025.

[60] bklemm@foley.com. Clinical Trials: FDA Publishes Draft Guidance on Diversity Action Plans. Foley & Lardner LLP. 2024. https://www.foley.com/insights/publications/2024/07/clinical-trials-fda-guidance-diversity-action-plans/. Accessed 3 Oct 2025.

[61] HRA and MHRA draft inclusion and diversity guidance. Health Research Authority. https://www.hra.nhs.uk/about-us/news-updates/hra-mhra-guidance-developing-and-submitting-inclusion-and-diversity-plan/. Accessed 3 Oct 2025.

[62] EMA adopts guideline on requirements for clinical-stage ATMPs. https://www.raps.org/news-and-articles/news-articles/2025/2/ema-adopts-guideline-on-requirements-for-clinical. Accessed 3 Oct 2025.

[63] MHRA - Implementation of ICH E6(R3) | Research Quality Association | RQA. https://www.therqa.com/news/mhra-implementation-ich-e6r3/. Accessed 3 Oct 2025.

[64] Commissioner O of the. Clinical trials guidance documents. FDA. 2025.

[65] Clinical Trials Transformation Initiative. CTTI recommendations: planning for successful trial recruitment. Durham (NC): Clinical Trials Transformation Initiative; 2016 (updated 2018 Jul). Available from: https://ctti-clinicaltrials.org/wp-content/uploads/2021/06/CTTI_Recruitment_Recs.pdf. Accessed 3 Oct 2025.

[66] Rare disease journey: understanding the barriers to trial participation. https://www.starkravinghealth.com/blog/the-rare-disease-journey-understanding-the-barriers-to-clinical-trial-participation. Accessed 3 Oct 2025.

[67] Advancing equity in rare disease trials: global realities, local solutions. https://www.pharmasalmanac.com/articles/advancing-equity-in-rare-disease-trials-global-realities-local-solutions. Accessed 3 Oct 2025.

[68] Newton W. Q&A: ethics of ultra-rare disease drug development and expanded access. Clinical Trials Arena. 2023. https://www.clinicaltrialsarena.com/interviews/ethics-ultra-rare-disease-drug-development/. Accessed 6 Oct 2025.

[69] New global guidance puts forward recommendations for more effective and equitable clinical trials. https://www.who.int/news/item/25-09-2024-new-global-guidance-puts-forward-recommendations-for-more-effective-and-equitable-clinical-trials. Accessed 3 Oct 2025.

[70] A primer on global regulatory guidance for diversity in clinical trials. Core Clinical Sciences. https://www.coreclinicalsciences.com/news/primer-global-regulatory-diversity-plans. Accessed 3 Oct 2025.

[71] England Rare Diseases Action Plan 2025: main report. GOV.UK. https://www.gov.uk/government/publications/england-rare-diseases-action-plan-2025/england-rare-diseases-action-plan-2025-main-report. Accessed 3 Oct 2025.

[72] Drug development for rare diseases: a case for patient-centricity, equity, and access to clinical trials | Journal of Rare Diseases. https://link.springer.com/article/10.1007/s44162-025-00069-y. Accessed 3 Oct 2025.

[73] Mitani AA, Haneuse S. Small data challenges of studying rare diseases. JAMA Netw Open. 2020;3:e201965. 10.1001/jamanetworkopen.2020.1965. 32202640 10.1001/jamanetworkopen.2020.1965

[74] Ferguson JYKMFEBRBKBN. Bridging the recruitment gap for socioeconomically disadvantaged groups in clinical trials | Applied Clinical Trials Online. 2025. https://www.appliedclinicaltrialsonline.com/view/bridging-the-recruitment-gap-for-socioeconomically-disadvantaged-groups-in-clinical-trials. Accessed 3 Oct 2025.

[75] Small population clinical trials – IRDiRC. https://irdirc.org/activities/task-forces/small-population-clinical-trials/. Accessed 3 Oct 2025.

[76] Gedela K, Wong R, Balendra S, Chita S, Jones H, Golombek R, et al. Embedding equity, diversity and inclusion processes within clinical trials and health and social care research. 2025. 10.1136/bmjopen-2024-091807. 10.1136/bmjopen-2024-091807 PMC11956285 40147995

[77] Patterson AM, O’Boyle M, VanNoy GE, Dies KA. Emerging roles and opportunities for rare disease patient advocacy groups. Ther Adv Rare Dis. 2023;4:26330040231164424. 10.1177/26330040231164425. 37197559 10.1177/26330040231164425 PMC10184204

[78] Merkel PA, Manion M, Gopal-Srivastava R, Groft S, Jinnah HA, Robertson D, et al. The partnership of patient advocacy groups and clinical investigators in the rare diseases clinical research network. Orphanet J Rare Dis. 2016;11:66. 10.1186/s13023-016-0445-8. 27194034 10.1186/s13023-016-0445-8 PMC4870759

[79] MBA MJL PhD; Abigail Dirks, MS; Nicholas Kikuchi; Neha Patel Cervantes; Ken Getz. An examination of the use of patient recruitment and retention tactics for global studies | Applied Clinical Trials Online. 2025. https://www.appliedclinicaltrialsonline.com/view/an-examination-of-the-use-of-patient-recruitment-and-retention-tactics-for-global-studies. Accessed 6 Oct 2025.

[80] Alsumidaie M. How to navigate rare disease clinical trial recruitment. The Clinical Trial Vanguard; 2024. Available from: https://www.genomemedical.com/wp-content/uploads/2025/02/How-to-Navigate-Rare-Disease-Clinical-Trial-Recruitment_The-Clinical-Trial-Vanguard_Oct-2024.pdf. Accessed 6 Oct 2025.

[81] Improving inclusion of under-served groups in clinical research: Guidance from INCLUDE project | NIHR. https://www.nihr.ac.uk/improving-inclusion-under-served-groups-clinical-research-guidance-include-project. Accessed 6 Oct 2025.

[82] Advancing equity in rare disease trials: global realities, local solutions. https://www.pharmasalmanac.com/articles/advancing-equity-in-rare-disease-trials-global-realities-local-solutions. Accessed 6 Oct 2025.

[83] Navigating CTIS: Setting up clinical trials under EU CTR. https://www.iconplc.com/insights/blog/2023/10/26/navigating-ctis-setting-clinical-trials-under-eu-ctr. Accessed 6 Oct 2025.

[84] UK Government unveils 2025 England rare diseases action plan to enhance research and treatment. 2025. https://bioresource.nihr.ac.uk/news/uk-government-unveils-2025-england-rare-diseases-action-plan-to-enhance-research-and-treatment/. Accessed 6 Oct 2025.

[85] Read “Exploring novel clinical trial designs for gene-based therapies: proceedings of a workshop” at NAP.edu. 10.17226/25712. 32730016

[86] Guideline on safety and efficacy follow-up and risk management of advanced therapy medicinal products - Scientific guideline | European Medicines Agency (EMA). 2018. https://www.ema.europa.eu/en/guideline-safety-efficacy-follow-risk-management-advanced-therapy-medicinal-products-scientific-guideline. Accessed 6 Oct 2025.

[87] EMA issues new guidelines for clinical development of advanced therapy medicinal products. MedPath. 2025. https://trial.medpath.com/news/5f802151287d8779/ema-issues-new-guidelines-for-clinical-development-of-advanced-therapy-medicinal-products. Accessed 6 Oct 2025.

[88] Advanced therapy medicinal products: regulation and licensing in UK. GOV.UK. 2025. https://www.gov.uk/guidance/advanced-therapy-medicinal-products-regulation-and-licensing. Accessed 6 Oct 2025.

[89] Gene Therapy Advisory Committee. Health Research Authority. https://www.hra.nhs.uk/about-us/committees-and-services/res-and-recs/gene-therapy-advisory-committee/. Accessed 6 Oct 2025.

[90] Chapman CR, Cripe TP, Bateman-House AS. Patient-centered long-term follow-up for gene therapies aligns with ethics and science. Mol Ther. 2025;33:2336–8. 10.1016/j.ymthe.2025.04.040. 40373770 10.1016/j.ymthe.2025.04.040 PMC12172254

[91] https://www.azenta.com ALS, Cretu S. Long-term follow-up in CGT: finding the “Just Right” approach. Azenta Life Sciences. 2025. https://www.azenta.com/learning-center/blog/the-goldilocks-goal-in-long-term-follow-up-for-cell-gene-therapies-finding-the-just-right-approach. Accessed 6 Oct 2025.

[92] Patient-centered long-term follow-up for gene therapies aligns with ethics and science. The Multi-Regional Clinical Trials Center of Brigham and Women’s Hospital and Harvard. https://mrctcenter.org/resource/patient-centered-long-term-follow-up-for-gene-therapies-aligns-with-ethics-and-science/. Accessed 6 Oct 2025. 10.1016/j.ymthe.2025.04.040 PMC12172254 40373770

[93] Bateman-House A, Cowley K, Fernandez V, Gilmor M, Hunt C, Nevoret M-L, et al. Lived experience of patients and caregivers in rare genetic neurological gene therapy clinical trials in children. Pediatr Neurol. 2025;163:46–9. 10.1016/j.pediatrneurol.2024.11.001. 39671832 10.1016/j.pediatrneurol.2024.11.001

[94] Roctavian | European Medicines Agency (EMA). 2022. https://www.ema.europa.eu/en/medicines/human/EPAR/roctavian. Accessed 6 Oct 2025.

[95] First gene therapy to treat severe haemophilia A | European Medicines Agency (EMA). 2022. https://www.ema.europa.eu/en/news/first-gene-therapy-treat-severe-haemophilia. Accessed 6 Oct 2025.

[96] CSL Behring’s HEMGENIX® (etranacogene dezaparvovec-drlb) demonstrates at three years post-treatment long-term durability, safety and greater bleed protection versus prophylactic treatment in people living with hemophilia B. Global Newsroom | CSL. https://newsroom.csl.com/2023-12-11-CSL-Behrings-HEMGENIX-R-etranacogene-dezaparvovec-drlb-Demonstrates-at-Three-Years-Post-Treatment-Long-Term-Durability,-Safety-and-Greater-Bleed-Protection-Versus-Prophylactic-Treatment-in-People-Living-with-Hemophilia-B. Accessed 6 Oct 2025.

[97] Ugoji C, Heidt J, Largent J, Bratton E, Hester L, Keshavarzi S, et al. Important tool in our rare disease toolbox: hybrid retrospective-prospective natural history studies serve well as external comparators for rare disease studies. Front Drug Saf Regul. 2024;4. 10.3389/fdsfr.2024.1418050. 10.3389/fdsfr.2024.1418050 PMC12443106 40979382

[98] Izem R, McCarter R. Randomized and non-randomized designs for causal inference with longitudinal data in rare disorders. Orphanet J Rare Dis. 2021;16:491. 10.1186/s13023-021-02124-5. 34814939 10.1186/s13023-021-02124-5 PMC8609847

[99] Shah MR, Powers E, Finot E. Long-term follow-up for gene therapies – innovative, patient-centered approaches. Insight brief. Durham (NC): IQVIA; 2021. Available from: https://www.iqvia.com/~/media/Biotech/PDFS/LI/Long-term-follow-up-for-gene-therapies. Accessed 3 Oct 2025.

[100] Patient-focused strategies for long-term follow-up in gene therapies. https://www.iqviabiotech.com/blogs/2024/04/patient-focused-strategies-for-long-term-follow-up-in-gene-therapies. Accessed 6 Oct 2025.

[101] Florez MI, Botto E, Kim JY. Mapping strategies for reaching socioeconomically disadvantaged populations in clinical trials. JAMA Netw Open. 2024;7:e2413962. 10.1001/jamanetworkopen.2024.13962. 38848069 10.1001/jamanetworkopen.2024.13962 PMC11161842

[102] Kim JY, Florez M, Botto E, Belgrave X, Grace C, Getz K. The influence of socioeconomic status on individual attitudes and experience with clinical trials. Commun Med. 2024;4:172. 10.1038/s43856-024-00586-9. 39237734 10.1038/s43856-024-00586-9 PMC11377822

[103] Quality By design. CTTI. https://ctti-clinicaltrials.org/about/ctti-projects/quality-by-design/. Accessed 6 Oct 2025.

[104] Quality by design in clinical trials under ICH E6(R3): emphasizing proactive quality. https://theqarp.com/blog_9. Accessed 6 Oct 2025.

[105] Rare diseases: UK framework. GOV.UK. 2021. https://www.gov.uk/government/collections/rare-diseases-uk-framework. Accessed 6 Oct 2025.

